# The Bit Scale: A Metric Score Scale for Unidimensional Item Response Theory Models

**DOI:** 10.1017/psy.2025.10071

**Published:** 2025-12-10

**Authors:** Joakim Wallmark, Marie Wiberg

**Affiliations:** Dept. of Statistics, USBE, Umea University, Sweden

**Keywords:** bit scale, bit scores, information, rescaling, scale transformation

## Abstract

In item response theory (IRT), the conventional latent trait scale ( 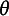 ) is inherently arbitrary, lacking a fixed unit or origin and often tied to specific population distributional assumptions (e.g., standard normal). This limits the direct comparability and interpretability of scores across different tests, populations, or model estimation methods. This article introduces the “bit scale,” a novel metric transformation for unidimensional IRT scores derived from fundamental principles of information theory, specifically surprisal and entropy. Bit scores are anchored to the properties of the test items rather than the test-taker population. This item-based anchoring ensures the scale’s invariance to population assumptions and provides a consistent metric for comparing latent trait levels. We illustrate the utility of the bit scale through empirical examples: demonstrating consistent scoring when fitting models with different 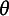 scale assumptions, and using anchor items to directly link scores from different test administrations. A simulation study confirms the desirable statistical properties (low bias and accurate standard errors) of Maximum Likelihood-estimated bit scores and their robustness to extreme scores. The bit scale offers a theoretically grounded, interpretable, and comparable metric for reporting and analyzing IRT-based assessment results. Software implementations in R (bitscale) and Python (IRTorch) are available and practical implications are discussed.

## 1 Introduction

**Figure 1:**
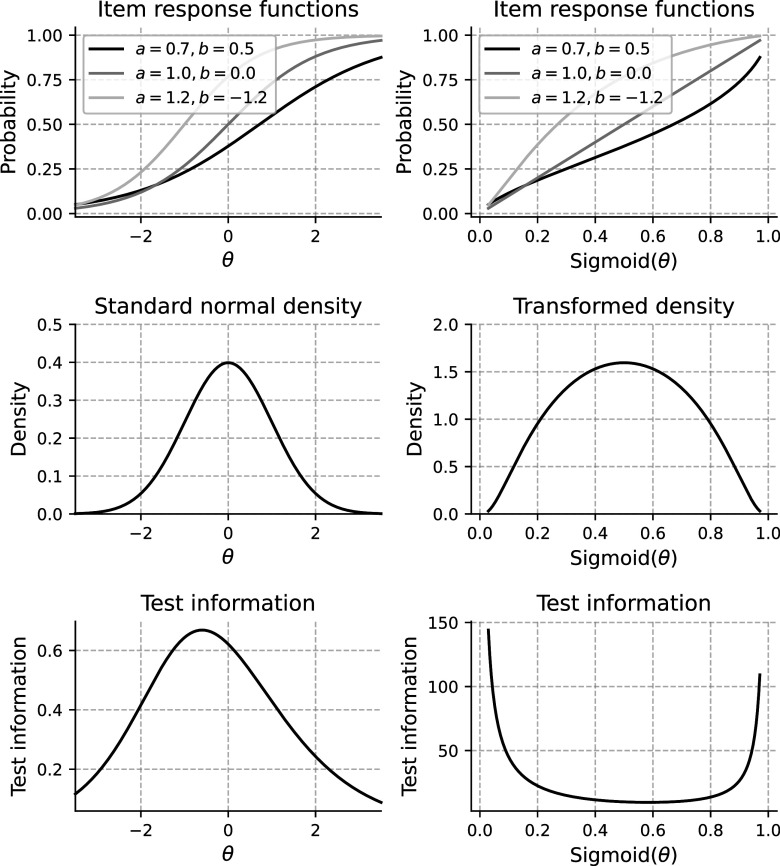
Item response functions, 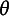 distributions, and test information curves before and after sigmoid transformation of 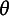 scale. *Note*: In the upper plots, *a* and *b* refer to the slope and difficulty parameters of the 2PL model as later described in Equation (1).

Item response theory (IRT) models (van der Linden, 2016) are commonly used when analyzing large-scale assessments and achievement tests. In IRT, the scale used for the latent variable 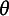 is inherently arbitrary. This means that it lacks a natural origin or unit (Lord, 1975). For reasons of statistical identifiability, interpretability, and computational convenience within estimation algorithms, it is common practice to assume 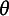 is continuous and follows a specific distribution, typically the standard normal distribution ( 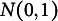 ). For example, estimation methods, such as marginal maximum likelihood (MML; Bock & Aitkin, 1981) and Bayesian approaches (Levy & Mislevy, 2017), are designed around such distributional assumptions. Because the scale is arbitrary, any monotonic transformation (one that preserves the rank order of individuals) can be applied without changing the model’s fundamental properties. Figure 1 demonstrates this principle by applying the sigmoid transformation to the original 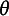 scale of a three-item test using the two-parameter logistic (2PL) model. Although the shape of the 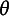 distribution changes, the relative ordering of test-takers and the original response probabilities are preserved, resulting in an equally valid representation of the IRT model. However, the slopes of the item response functions (IRFs) and, as a direct consequence, also the test and item information curves (Hambleton & Swaminathan, 1985, Chapter 6) change profoundly. As a result, distances on the latent trait scale only have meaning in relation to other people in the population from which the sample of test takers used to fit the model was drawn. These issues have been known for a long time and have been discussed previously by Lord (1975).

The overall aim of this article is to introduce a scale transformation, which we refer to as the bit scale transformation to circumvent these issues. Instead of arbitrarily deciding on a distribution and tying the latent trait scale to the test taker population, bit scales are metric scales tied to the test items. To define bit scales, we will use ideas from information theory (Cover & Thomas, 2006; Shannon, 1948). The utility of bit scales will be illustrated through two empirical examples and a simulation study.

Another alternative to the 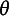 scores includes D-scores (Dimitrov, 2024), which are scores computed from the test takers’ response vector, and are weighted by the expected difficulties of the items. The expected difficulty of an item is obtained analytically from its IRT parameters. Additionally, the use of surprisal arc length has been proposed (Ramsay et al., 2020, 2024). A limitation, however, with using arc length is that it is not defined when the probability of a correct response is zero. This situation can occur when we use an IRT model, which gives small or zero probabilities, which is the case for many commonly used IRT models. Examples include the 2PL model (Birnbaum, 1968) and the generalized partial credit (GPC) model (Muraki, 1992) introduced in the next section. The proposed approach avoids this problem and can thus be seen as a refinement of the use of arc length.

The rest of this article is structured as follows. In the next section, IRT is briefly summarized, including the two models we will use. Section 3 provides a brief introduction to information theory, and surprisal and entropy are described. The fourth section introduces bit scores and bit scales, followed by the fifth section, which contains two empirical illustrations with real test data and a software note. This is followed by a simulation study where bias, standard error (SE), and root mean squared error (RMSE) are examined for both binary and polytomously scored items. The article ends with a discussion with some concluding remarks that includes suggestions on how bit scales can be used in practice.

## 2 Item response theory

IRT provides a framework for modeling the relationship between an individual’s latent trait (e.g., ability and attitude) and their responses to items on a test or questionnaire (van der Linden, 2016). Unlike classical test theory, which primarily focuses on total test scores, IRT examines responses at the item level. A central concept in IRT is the IRF, which mathematically describes the probability of a specific response to an item given the level of the latent trait.

We consider a test comprising *J* items, indexed by 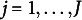 . The response of an individual to item *j* is denoted by 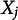 and is considered a random variable. We denote the possible item responses for item *j* as 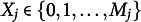 and the probability to respond with 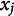 at a given ability level 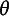 (the IRF) as 
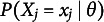
 . Several specific IRT models exist, differing in the functional form of the IRF and the item parameters used. Below, we define the models relevant to this article.

For items scored as correct/incorrect, the most common models belong to the logistic family. The 2PL model (Birnbaum, 1968) defines the probability of a correct response ( 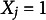 ) to item *j* given 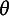 as 
(1)

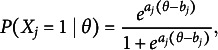

where 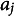 is the discrimination parameter, indicating how well the item differentiates between individuals with different trait levels (higher 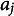 means steeper slope of the IRF), and 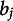 is the difficulty parameter, representing the trait level required for a 50% chance of a correct response. The probability of an incorrect response is 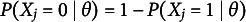 .

For items with more than two ordered response categories, the GPC (Muraki, 1992) Model models the probability of responding with 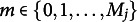 on item *j* as 
(2)

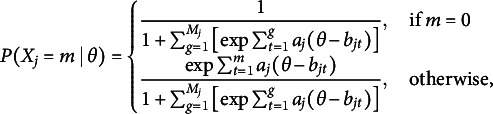

where 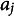 is the item discrimination parameter, and 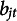 are threshold parameters, defining the points on the 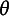 scale at which the probability of responding with a score of *t* surpasses that of responding with 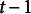 .

The IRT models considered in this study, as well as most unidimensional IRT models, rely on the following fundamental assumptions (e.g., Hambleton & Swaminathan, 1985, Chapter 2):

- *Unidimensionality:* The model assumes that only a single latent trait 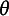 underlies the individuals’ responses to the set of items.
- *Local independence:* Given an individual’s latent trait level 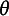 , their responses to different items are statistically independent. That is, 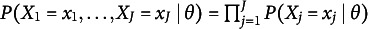 .
- *Monotonicity:* As the latent variable 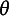 increases, the probability of a higher score on an item also increases.

## 3 Information theory

Information theory, originally proposed by Shannon (1948) and later discussed in Cover and Thomas (2006) and Stone (2022), is the mathematical representation of the transmission of messages through a communication system. In a test setting, each possible item response can be seen as a message and the communication system is the test as noted by Ramsay et al. (2024)). In information theory, an important concept is self-information, or the term surprisal coined by Tribus (1961). Surprisal is derived from the probability of a particular event occurring from a random variable. The transformation 
(3)

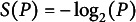



converts a probability *P* into surprisal, and thus 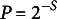 converts a surprisal into probability. Thus, probability and surprisal can be viewed as two different ways to look at a response option. Due to the logarithmic transformation, surprisal ranges from 0 to *∞*. For an event that is certain to occur, with a probability of 1, the surprisal is 0, there is no surprise, as we know that the event will occur. A rare event yields more information and thus a higher surprisal value, and if 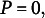 the surprisal is 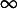 . As surprisal has metric properties, we can add, subtract, and multiply it with positive constants.

The surprisal transformation is not new, as it has been used in many areas in statistics, such as in the definitions of log-odds transform, negative log likelihood, deviance (Kullback, 1959), and in mathematical psychology, it is used in the theory of choice (Luce, 1959). Surprisal satisfies the requirements of a ratio scale (Stevens, 1946): it possesses an absolute zero (corresponding to an event with probability 1), is nonnegative, and is additive for independent events (Shannon, 1948). Also, using surprisal instead of probability often simplifies computations, as the occurrence of multiple independent events results in multiplication when dealing with probabilities, but becomes addition after being transformed into surprisal.

Entropy quantifies the average level of uncertainty in a random variable’s possible outcomes. It is the expected surprisal. For an item *j* with possible response options 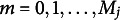 , and a test taker with latent trait *θ* we can define entropy as 
(4)

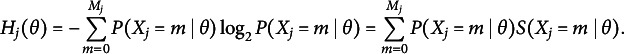



When 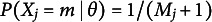 for all possible response options *m*, the entropy is maximized. Entropy can be seen as a measure of the lack of structure and when every response option probability except one is zero, it reflects certainty (Ramsay et al., 2024).

## 4 Bit scales

The surprisal transformation in (3) is measured in bits and quantifies uncertainty. Unlike probability, it has metric properties. The term “bits” originates from the field of digital computing and “Bit” is an abbreviation for “binary digit.” In the context of information theory, one bit represents the amount of uncertainty (or information gain) in each outcome of a random variable with two equally likely outcomes, like a fair coin flip. Just as the expectation of a random variable measured in meters inherits the same unit of measurement as the original random variable, entropy inherits the bit unit and quantifies the expected information gain across all outcomes.

The 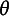 scale is fundamentally ordinal. However, it is evident in Equation (4) that as *θ* changes, *H*_*j*_(*θ*) also changes. We leverage this relationship to construct a new scale for the latent trait based on the cumulative change in entropy. Since entropy, as the expected surprisal, is measured in bits (a ratio-level unit), the cumulative measure of its change will also be on a ratio scale. We define the item bit score, located on the item bit scale, as the total distance traveled in *H*_*j*_(*θ*) after moving from the minimum *θ*, *θ* ^(0)^, to the test taker’s estimated *θ*, 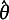



(5)

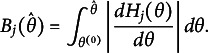



**Figure 2:**
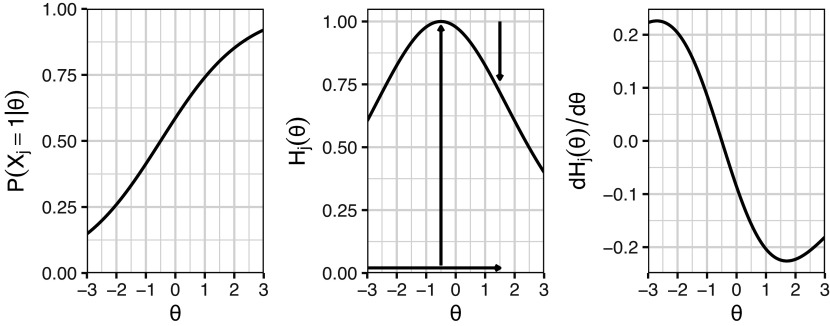
Example IRF to the left with its corresponding entropy curve in the middle, and the derivative of the entropy curve to the right. The vertical arrows in the entropy plot show the distances added up to compute the item bit score for a test taker with an estimated 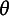 of 1.5.

The procedure for calculating these bit scores is depicted in the middle plot of Figure 2. Using an example of a test taker with an estimated 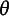 of 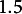 on the first item from Figure 1, we can trace the entropy changes starting from the minimum 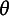 . As indicated by the vertical arrows, the entropy initially increases by 1 bit (from when 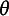 approaches 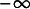 ) and subsequently decreases by 0.28 bits. The final bit score is obtained by adding the absolute magnitudes of these changes, resulting in 1+0.28=1.28 bits. The entropy curve measures the amount of uncertainty in the item response at each level of 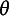 (Stone, 2022), and this uncertainty of course changes as 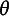 changes. The entropy bit score thus measures the change in item response uncertainty as a test taker progresses across the latent trait continuum. A possible interpretation would be that, on average, a test taker with 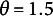 has obtained 1.28 out of the total 2 bits of latent trait information contained within the item.

As IRT models typically assume local independence, we can use that assumption to add the item bit scores together to get a bit score for the whole test based on the item bit scores as a function of *θ* as follows: 
(6)

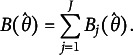



Both item bit scores and test bit scores are monotonic one-to-one functions of 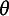 , and like any other such transformation, they can be used as a valid replacement for 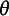 .

One might naturally question how the ordinal 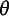 scale can be transformed into a ratio scale. The justification lies in a fundamental change of the variable being measured: the bit scale does not quantify the latent trait as a static coordinate, but rather quantifies the *accumulated resolution of uncertainty* provided by the test items regarding that trait. While the coordinate 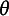 is arbitrary, the item response surprisals 
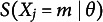
 and their corresponding entropy curves are absolute. Consequently, the bit scale satisfies the requirements of a ratio scale (Stevens, 1946):

- *Absolute origin:* The scale is mathematically anchored at the lower boundary of the integration, 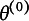 . A bit score of 0 implies that, relative to this starting point, the test has not yet distinguished the test-taker from the minimum measurable state. There is an absolute absence of accumulated information change.
- *Standardized unit:* Unlike the standardized units of 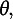 which depend on population variance, the “bit” is a fixed unit of information derived directly from probability. One bit of entropy corresponds to the uncertainty of a binary event with 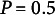 . This unit is invariant across items, tests, and populations.
- *Equality of intervals and ratios:* Because the score is defined as an integral (Equation (5)), it is inherently additive. A test taker with a score of 4 bits has traversed exactly twice the informational distance of a test taker with 2 bits.

Thus, scores on the bit scale are naturally comparable between different populations as long as there are common items in the tests taken by each population. While 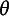 is typically tied to the test taker population and assumed to be standard normally distributed, bit scores are tied to the test items and invariant to the population taking the test. A test bit score of 0 means that the measured latent trait is at the minimum or below what can be measured with the items in the test, while a maximum score (all bits from all items) means that the test taker is at the maximum or above what the test can measure.

The maximum score corresponds to the total accumulated entropy change across all items. In the simplest case, for a binary item where the probability of a correct response goes from 0 to 1 (assuming a model with no guessing probability incorporated), the entropy increases from 0 to 1 bit (at 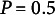 ) and then decreases back to 0. The total bit score for such an item would be 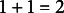 bits. Thus, for a test consisting of *J* binary items, the bit scale ranges from 0 to 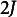 . Extending the interpretation from the single item case, the total bit score represents the aggregate resolution of uncertainty across the entire test. For example, consider a hypothetical test consisting of 10 items similar to the one in Figure 2. Since each item has a total information capacity of 2 bits, the maximum possible score for the test is 20 bits. In this context, a test taker with a test bit score of 12.8 has obtained 12.8 out of the 20 bits of latent trait information contained within the test structure. This score reflects the cumulative informational distance the test taker has traversed along the measurement scale.

For more complex cases with polytomously scored items or when there is guessing incorporated in the chosen IRT model, the maximum bit score is harder to know in advance. In such cases, bit scores may appear a bit arbitrary, especially for those unfamiliar with the methodology. In such cases, the entire bit scale can be linearly rescaled to range from 0 to 100 or 0 to 1, which changes the score interpretation to percentages of test information (bits) attained at a given score. Next, we derive the SE and asymptotic distribution for ML-estimated bit scores.

### 4.1 Standard errors and asymptotics of bit scores

We denote the true 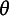 score of a test taker as 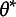 and their ML estimate as 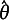 and make the assumption that the estimated IRFs are the true ones. It is well established that the estimator 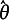 is consistent for 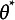 and that, asymptotically, 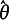 follows a normal distribution with mean 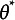 and variance 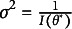 (van der Vaart, 2000, Chapter 5.5). Here, 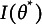 represents the Fisher information evaluated at 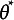 , defined as 

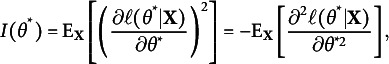

where 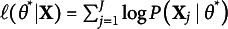 is the log-likelihood of 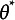 given response vector 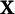 , available from the model IRFs, and the expectation is taken over all possible values of 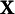 . That is, we have 
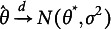
 . Thus, it follows from the delta method (van der Vaart, 2000, Chapter 3) that 
(7)

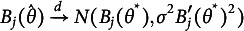



(8)

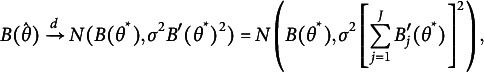

where 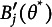 is the derivative of 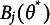 . By the Fundamental Theorem of Calculus, 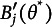 is the integrand of 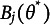 evaluated at the upper integration limit 

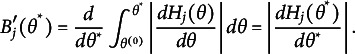

From Equation (7), we see that for each item bit score, the SE of 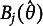 is simply the SE of the ML estimate of 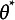 multiplied by 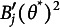 , the magnitude of the slope of the item entropy curve at 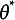 .

In IRT, item information and test information are commonly used to quantify the accuracy with which an item or test measures an individual’s ability level (Hambleton & Swaminathan, 1985, Chapter 6). Bit scale test and item information can also be retrieved by asymptotic theory. Under standard regularity conditions, these information metrics can be computed from the reciprocal of the variance of 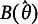



(9)

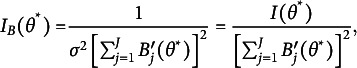



(10)

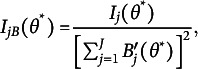

for test and item information, respectively, where 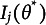 is the Fisher information about 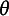 given by item *j* at 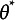 . Note that all 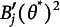 are still used for bit scale item information, since the scale itself relies on all items. 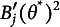 is the information from the item response about the test bit score. This also means that the item information sums up to the test information just like with the traditional 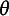 scale. That is, we have 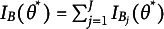 since 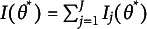 . Since the denominator in Equation (9) is simply a scaling factor derived from the bit scale transformation itself, it is independent of a specific test taker’s response pattern. Thus, the observed Fisher information can be written as 
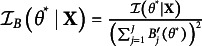
 , where 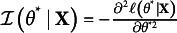 is the observed Fisher information on the 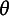 scale for a given response vector 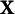 .

### Relationship to other functions of 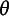


4.2

Fisher information is a function of 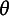 just like the bit score itself. However, it is not a monotonic transformation of 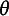 , and thus not a valid score scale for an IRT model. Fisher information is a local measure of precision often used to construct error bands, while the bit scale is a cumulative metric that defines the score scale itself.

One should also consider the similarities between the bit score and expected sum score at a given 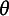 , defined as 

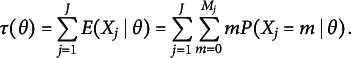




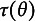
 is, just like the bit score, a valid 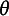 transformation tied to the test. However, the unit is different (bits vs. observed test score). The expected sum score accumulates the *magnitude* of the response probabilities at a given 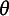 weighted by predefined item scores. In contrast, the bit score is invariant to the predefined item scores (the magnitude of *m* in the above equation) making it less sensitive to predetermined notions of which items are more or less important. Instead, the bit score accumulates the change in bits of uncertainty across the 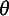 scale. As a consequence, non-informative items without much slope in their curves or items that are easy to guess correctly can have a huge impact on the expected sum score. With the bit score, the impact of such items can be greatly reduced. As an extreme example, an item with a flat IRF (where the probability to respond correctly is constant) contributes a constant amount to the expected sum score (provided the probability is nonzero), but the same item contributes nothing to the bit score.

## 5 Two empirical illustrations

In this section, we provide two real data examples to illustrate the usefulness of bit scales. In the first example, we use data from the Swedish national mathematics test for high school students to show how bit scales can be used for model comparison when fitting models to the same data but with fitting algorithms using different assumptions about the 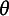 distribution. In the second example, we use data from the Swedish Scholastic Aptitude Test (SweSAT) to illustrate how bit scales can be used directly to link latent trait scores between IRT models fitted to different test forms with different populations when there are common (anchor) items available.

### 5.1 Bit scores when latent trait scale assumptions differ

The national mathematics test used in this example is a key component of the Mathematics 3c course in Swedish high schools. Taking this course is mandatory for students on the natural science and technology tracks, although it is available as an optional course for others. Administered at the end of the term, the outcome of this test carries considerable weight in determining the student’s final grade for the course. It features several different types of items, each contributing a varying amount of points to the total score. Summary statistics from the utilized dataset can be found in the corresponding column in Table 1.

**Table 1:** Summary statistics for each test form

	National	SweSAT			
	Mathematics	Main		Anchor	
Year	2019	2013	2014	2013	2014
Sample size	1,401	2,469	2,859	2,469	2,859
Number of items	28	80	80	40	40
Total score	58	80	80	40	40
Mean	25.54	40.94	40.12	17.71	17.56
Standard deviation	12.62	13.34	12.88	7.13	7.06
Skewness	0.15	0.32	0.35	0.6	0.56

**Figure 3:**
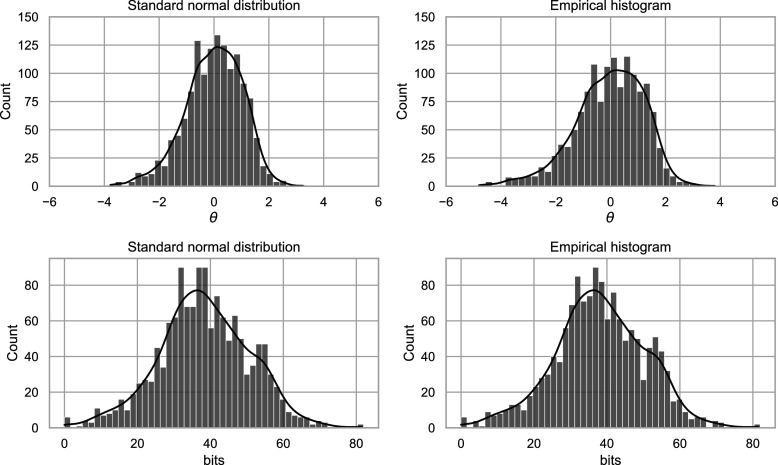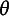 and bit score distributions for GPC models fitted with and without constraints on the 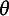 scale.

The GPC model (Equation (2)), chosen for its applicability to polytomously scored partial credit items, was fitted using two variants of MML implemented in the mirt package (Chalmers, 2012). One under the assumption of a standard normal latent trait distribution, and the other using the empirical histogram estimation method (Bock & Aitkin, 1981). The resulting score distributions from each model are shown in Figure 3 on both the bit and 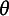 scales. The empirical histogram 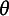 distribution has a larger variance (1.55) than its counterpart under the standard normal assumption (1.06). However, after transforming both scales to the bit scale, the distributions are more similar, and the score ranges are close to identical.

**Figure 4:**
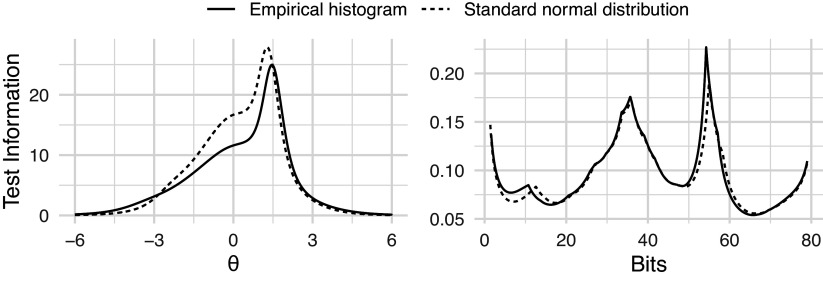
Fisher information for GPC models fitted with and without constraints on the 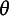 scale. Information is represented on both the 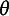 and the bit scales.

We also note in Figure 4 that the test information curves differ substantially depending on the chosen scale. On the 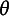 scale, the information curve is unimodal. This is largely a consequence of the fitting algorithm and the assumption of a standard normal distribution, which tends to compress measurement precision into a single smooth peak. In this case, the information is highest for above average test takers.

In contrast, the bit scale information curve exhibits multimodality. This shape is not an artifact but a reflection of the non-linear stretching of the scale defined by the specific items in the test. Because the bit scale expands in regions of high entropy change (high discrimination) and compresses elsewhere instead of being arbitrarily defined, it reveals a more granular structure of the test’s measurement precision. In this specific example, the peaks in the bit scale information curve (Figure 4) align with the peaks observed in the bit score distribution (Figure 3). Just like the 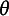 scale, the bit scale also suggests that the test is most informative for test takers above the population average. However, it also reveals distinct clusters of examinee scores and information that was hidden by the arbitrary standard normal assumption of the 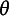 scale.

Therefore, while the 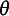 scale imposes a unimodal structure, the bit scale reveals that measurement precision for this specific test form is not uniform, but rather concentrated in specific zones defined by the item set. If items were added or removed, the bit scale—and consequently the shape of the information curve—would adapt to reflect the new measurement structure.

### 5.2 Equating IRT models with bit scales using anchor items

Bit scales can be used directly to compare latent trait scores between different IRT models when there are common (anchor) items available. We use data from two administrations of the SweSAT to illustrate this. The SweSAT is a binary-scored multiple-choice test with a quantitative and a verbal part that are equated separately. Each part contains 80 items and either a verbal or a quantitative anchor test with 40 items is given to a smaller number of test takers for the purpose of equating. Here, we are only considering the verbal part. Refer to Table 1 for summary statistics.

Since bit scores are tied to the test items, we can compute bit scores using only the items that are commonly available on both test administrations to provide a common bit scale between models even if test takers from different populations took each test form. For scoring, the 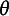 scores should still be estimated as accurately as possible using all items in the test, but then the bit scores should be computed using only the anchor items for a given 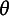 . We fitted three separate 2PL models using MML. The first two models utilized the complete datasets from the 2013 and 2014 test administrations, respectively. We observed that these populations exhibited similar ability distributions. Therefore, to provide a clearer demonstration of bit scale utility for contrasting ability groups, we constructed a third dataset. This involved sampling 1,000 test-takers from the 2014 administration, with selection probabilities weighted by the square of each test taker’s sum score relative to the total sum of squared scores, effectively creating a higher-ability sample. The third 2PL model was fitted to this specific sample.

**Figure 5:**
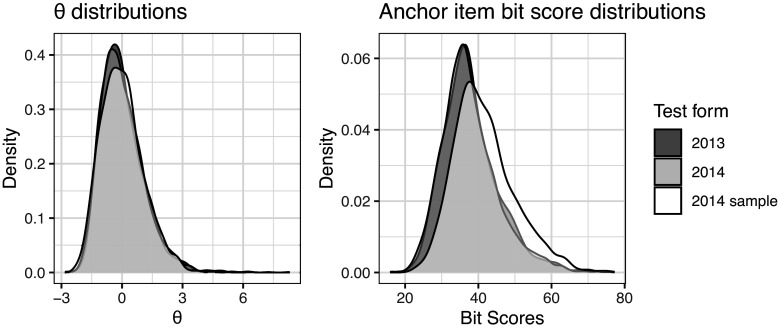
Latent score distributions.

The score distributions on both the 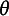 and bit scales (computed using only the anchor items) are shown in Figure 5 for all models. On the 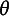 scale, all three curves are, as expected, close to standard normally distributed by assumption and the chosen fitting algorithm, though there is not a perfect overlap. On the bit scale, we see that the full dataset distributions are overlapping, but the more able sample has a larger portion of high scorers than the other samples. Since the bit scores use the same items, they are on the same scale and directly comparable.

**Figure 6:**
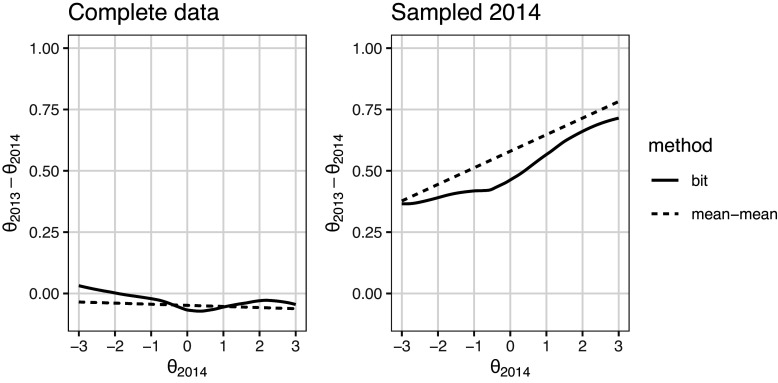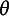 score transformation utilizing anchor item computed bit scores contrasted against the mean–mean linking method.

Figure 6 shows the transformation of a sequence of 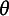 scores on the 2014 model scales. The anchor item bit scores were first computed using the 2014 dataset model. The scores were then converted to the 2013 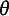 scale using the inverse of the bit score transformation with the 2013 dataset model. The resulting transformations are contrasted against the commonly used mean–mean linking (Kolen & Brennan, 2014, Chapter 6.3) for comparison. In mean–mean linking, a constant, derived from the ratio between the average discrimination parameters of the common items on the two forms, adjusts one form’s item parameters to the other’s scale. This results in a linear scale transformation. Since the bit scale method allows for non-linear scale relationships, it should better capture the relationship between the scales when it is not strictly linear.

When the complete dataset from 2014 was used, both methods suggest that the test taker groups are very similar in ability, and the transformation in Figure 6 is close to being one-to-one, as shown in the plot on the left. When the more able sample of 2014 was used to fit the 2014 model, both methods agree that the equivalent scores on the 2013 scales are somewhat higher, especially at the upper end of the 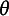 scale.

## 6 Simulation study

A simulation study was conducted to examine the asymptotic properties of bit scores and evaluate the bit score SEs derived in Section 4.1. Data were generated using the 2PL model. The item parameters were randomly sampled. Discrimination parameters were sampled from a lognormal distribution with mean 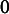 and variance 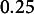 while difficulty parameters were sampled from a standard normal distribution. These distributions are commonly used in other simulation studies (Feinberg & Rubright, 2016). We examined test forms with 20, 40, 80, and 160 items. The simulation procedure is described in Algorithm 1. Note that the item parameters (and thus the IRFs) are assumed to be known and fixed throughout all iterations. This is similar to typical 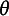 estimation methods, such as EAP, MAP, and ML for which the SEs are computed conditional on a given model. To analyze the behavior of the SE across the ability scale, we used a grid of evenly spaced true 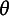 values. This grid is not intended to represent a realistic population distribution; however, this is not necessary since the item parameters were fixed a priori and not estimated from simulated population data. The R package mirt (Chalmers, 2012) was used to fit the models and estimate the 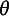 scores. The bitscale R package (Wallmark, 2025a) was used to estimate the bit scores. The simulation code is available on Github at https://github.com/joakimwallmark/bit-scale-simulation.

**Figure:**
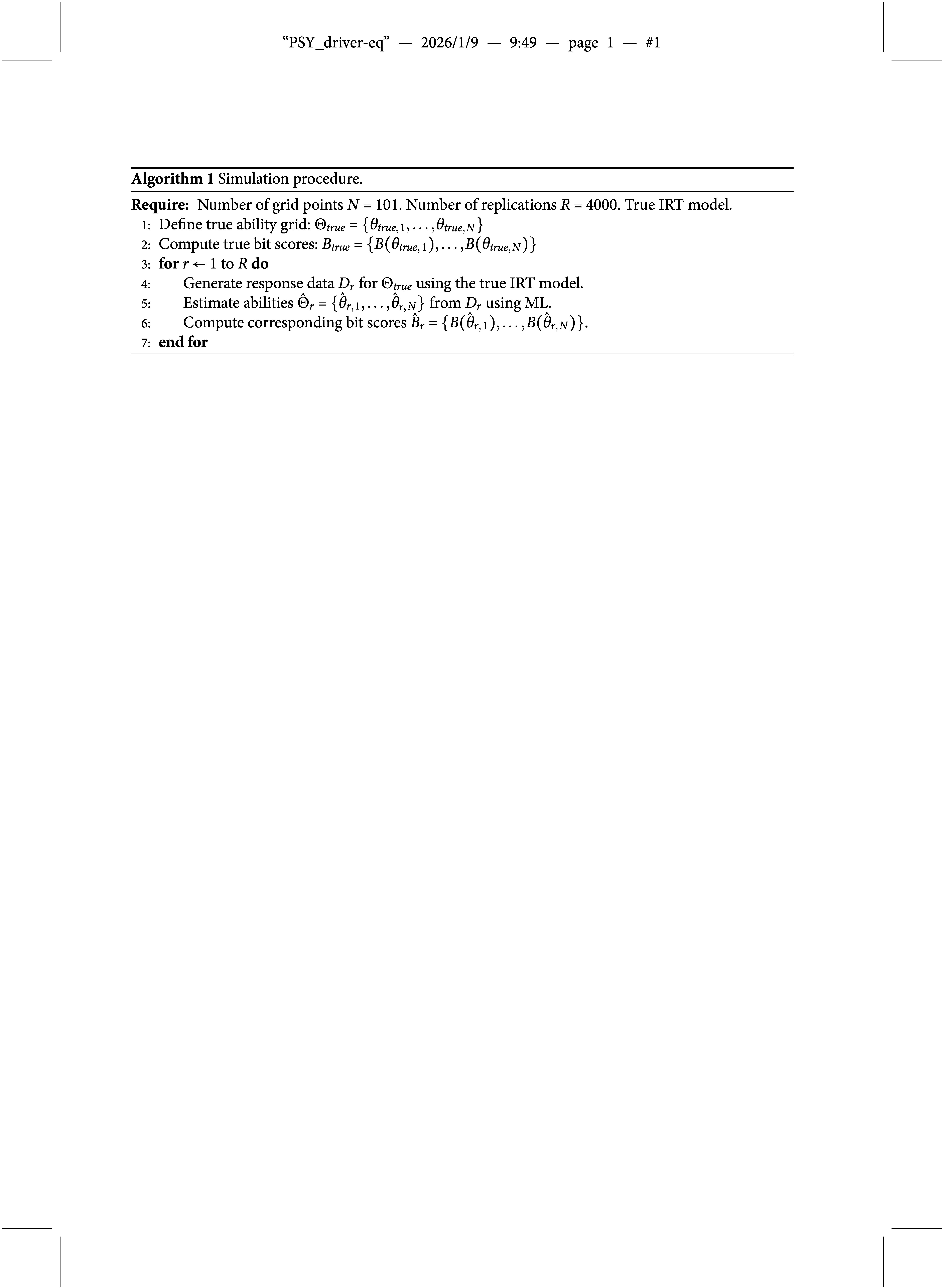

### 6.1 Evaluation measures

We used bias, SE, and RMSE as evaluation measures. Bias was calculated for ability and bit scores, respectively, as 

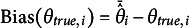

and 

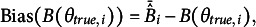



where 
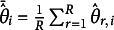
 and 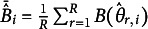 are the mean estimates of the 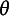 and the bit score, respectively. The 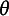 and bit score SE were obtained from 

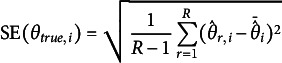

and 

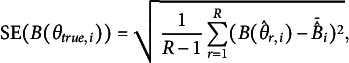

respectively. Finally, the 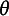 and bit score RMSE were computed using 

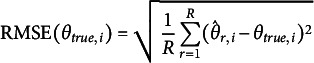

and 

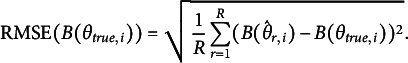



### 6.2 Simulation study results

**Figure 7:**
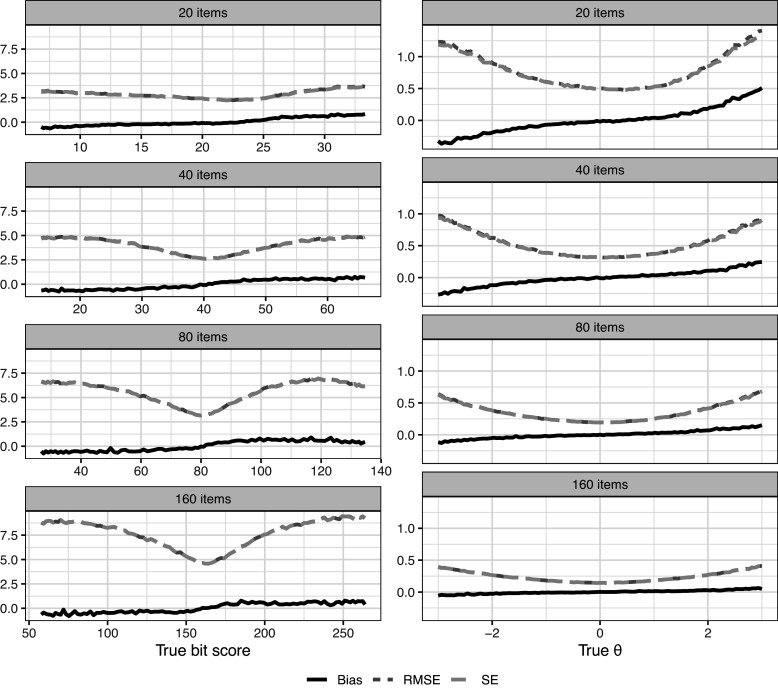
Bias, RMSE, and SE across the bit and 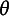 scales. Note that the SE and RMSE curves are close to overlapping due to the small bias in most settings.

Figure 7 shows bias, SE, and RMSE over the entire score scale. Of course, magnitudes cannot be directly compared because we are on different scales between plots. It is well known that a total score of zero or a perfect score results in 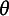 estimates of negative/positive infinity when using ML for score estimation. Because of this, estimated 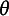 scores of negative and positive infinity were arbitrarily set to 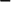 6 and 6, respectively, to be able to calculate the metrics for the 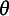 estimator. This adjustment is not needed for the bit scores. Despite this adjustment, it is clear that there is some substantial bias in absolute terms when using the 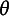 scale in scenarios with fewer items. Note, however, that the distances do not actually mean much because of the arbitrary nature of the 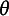 scale. A one unit increase in 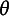 simply means one standard deviation in the arbitrarily chosen population distribution, but it says nothing about how much a respondent has actually improved on the test. This makes the bit scale a more natural choice when evaluating latent trait estimator performance in IRT settings using distance metrics, such as bias, SE, and RMSE due to the metric properties of the bit scale. It also performs well in situations like this without adjustment, where 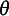 estimates can end up being infinitely small/large. The bias is also relatively small compared to the SEs in all settings. It should be noted that while the expected sum score is also a valid transformation of 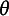 that avoids infinite estimates, its bias, SE, and RMSE are not directly comparable to the bit scale due to the difference in units (score points vs. bits).

**Figure 8:**
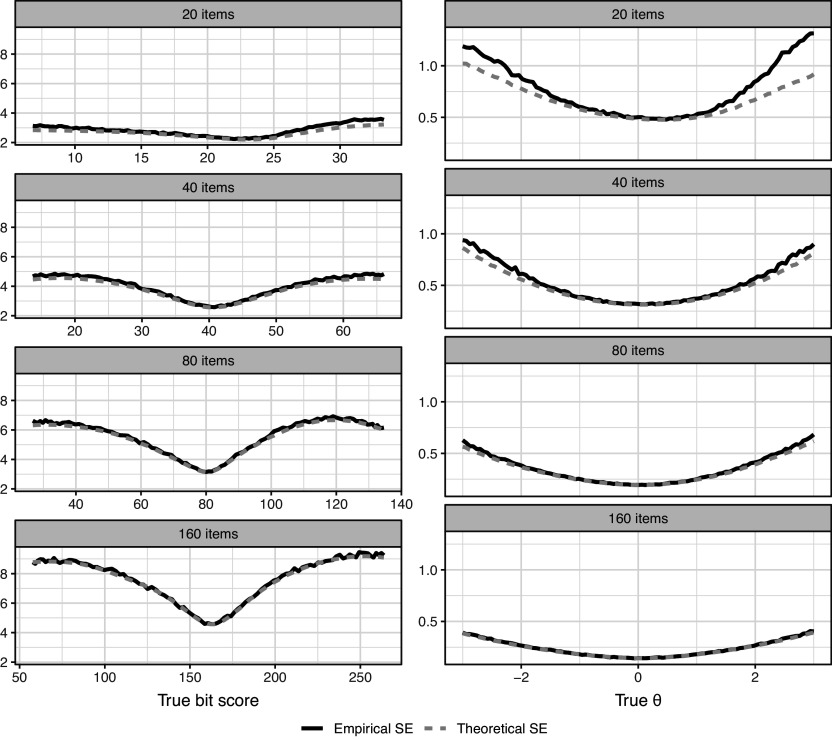
Simulation evaluation of theoretical SEs for ML-estimated bit and 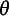 scores.

Figure 8 compares the empirical and theoretical SEs for the scales. Theoretical SEs for perfect/zero scores are undefined and were thus ignored in the simulations. We see that for both scale transformations, the theoretical SEs slightly underestimate the actual SEs with fewer items, but become more accurate as the number of items increases. This is expected from asymptotic theory and the derivations given in Section 4.1. The bit score SEs are larger when more items are used since each item increases the length of the entire scale. If one prefers a fixed length, the bit scale could be linearly rescaled to span, for example, from 0 to 100 as mentioned previously in Section 4. The interpretation would then be the percentage of bits of information within the test that a test taker has attained at a certain score.

## 7 Discussion

In this article, we introduced the bit scale, a novel metric transformation for latent trait scores derived from unidimensional IRT models. The primary motivation was to address the inherent arbitrariness of the traditional 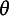 scale, whose interpretation is often tied to specific population assumptions (typically 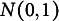 ) and on which distances are ambiguous (Lord, 1975). By leveraging fundamental concepts from information theory, specifically surprisal and entropy (Cover & Thomas, 2006; Shannon, 1948), the bit scale provides a score metric anchored to the properties of the test items rather than the distribution of test-takers.

The bit scale approach builds on related ideas using information-theoretic concepts by Ramsay et al. (2024), but avoids calculation issues when surprisals approach infinity (response probabilities approach zero), which occur with many IRT models. Furthermore, adding a single item to a test results in a fixed and predictable increase in the length of the score scale. This is not the case with the Ramsay et al. (2024) methodology where arc length is utilized for scoring. This additive nature of the bit scale should arguably also make bit scale score calculations more intuitive and easier to grasp. Furthermore, just like with arc length scores (Ramsay et al., 2024) methodology and the D-scoring approach by Dimitrov (2024), the bit scale is bounded and positive, making it more intuitive for the general public for which concepts, such as negative scores and scores approaching infinity may be confusing and hard to grasp. As mentioned in Section 4, the bit scale can also be rescaled to range from 0 to 100 or 0 to 1 if one prefers, which changes the score interpretation to percentages of test information (bits) attained at a given score.

Our proposed bit score, defined in Equations (5) and (6), quantifies the cumulative change in expected item surprisal (entropy) along the latent trait continuum. This construction yields a scale with metric properties and an absolute zero, where units are measured in “bits.” A key advantage highlighted is that distances on the bit scale have a consistent meaning related to the uncertainty resolved by responding to the test items, regardless of the underlying 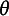 scale’s distribution. This was illustrated in the first empirical example (Section 5.1), where fitting a GPC model with and without a distributional assumption resulted in different 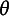 distributions but highly comparable bit score distributions (Figure 3). Furthermore, the Fisher information curves commonly utilized in IRT analysis lack consistency on differently scaled 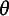 axes (Figures 1 and 4). The bit scale resolves this issue by providing a metric scale on which IRF slopes, and in turn also Fisher information, are more meaningful, arguably providing a more stable representation of measurement precision across the range of ability.

The second empirical illustration (Section 5.2) demonstrated the practical utility of bit scales for equating or linking latent trait scores from different test forms administered to potentially different populations, provided common anchor items exist. By computing bit scores based solely on the anchor items, we established a common, item-anchored metric enabling direct comparison of latent trait levels across the 2013 and 2014 SweSAT administrations, even when simulating a population shift (Figure 5). This offers a conceptually straightforward alternative or complement to traditional linking methods (e.g., mean–mean and characteristic curve methods (Kolen & Brennan, 2014)), particularly as it naturally handles potential non-linear relationships between scales induced by model differences or population variations, as shown in Figure 6. Future research should, however, more thoroughly examine the efficiency of the bit scale linking method against other approaches.

The simulation study (Section 6) investigated the statistical properties of ML-estimated bit scores. The results indicated that bit score estimates exhibit low bias, especially with longer tests, and behave consistently with asymptotic theory (Figure 7). The derived SEs (Section 4.1), based on the delta method, provided a good approximation of the empirical SEs, particularly for longer tests (Figure 8), validating their use for quantifying uncertainty in bit scores. Notably, the bit scale transformation naturally handles cases with perfect or zero scores, where ML estimates of 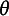 diverge to 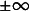 , offering a practical advantage in score estimation and evaluation across the entire ability range. The availability of implementations in R (bitscale) and Python (IRTorch) facilitates the adoption and further exploration of bit scales by researchers and practitioners (Wallmark, 2025a, 2025b).

Despite the promising characteristics of the bit scale, some limitations and areas for future research warrant discussion. First, the derivation of SEs in Section 4.1 assumes the item parameters (and thus the IRFs) are known without error. While the bit scale is designed to be invariant to population assumptions, its stability is still contingent on the precision of the underlying item parameter estimates. This uncertainty is not propagated into the bit score SEs presented here. This problem is not unique to the bit scores, and most 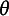 estimation methods also make the same assumption, such as EAP, MAP, and ML implemented in the mirt package (Chalmers, 2012). Future work could explore methods that incorporate item parameter uncertainty into the variance estimation of bit scores (Yang et al., 2012). Furthermore, evaluating the metric stability of a fitted model, as proposed by Feuerstahler (2022), could provide a valuable diagnostic tool to assess the quality of the bit scale itself due to uncertainty of the item parameters.

Second, the computation of bit scores involves numerical integration (Equation (5)), which, while straightforward for common IRT models using standard software, might become more complex for highly intricate models or require careful implementation choices regarding the integration grid ( 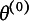 and density). Third, while “bits” provide a theoretically grounded unit, practitioners and test-takers accustomed to norm-referenced scores (like z-scores or T-scores) may require guidance and illustrative examples to fully internalize the meaning and interpretation of bit scores. One should note that utilizing the bit scale for some analysis does not exclude the use of the 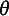 scale, and they can of course be used in conjunction.

Further investigation into the relationship between bit scale information (Equation (10)) and traditional Fisher information under various scaling conditions would be valuable. Comparing the performance of bit scale-based linking with established equating methods across a wider range of data conditions (e.g., different anchor test lengths, population differences, and model misfit) would also be beneficial. Exploring the application of bit scales in contexts like computerized adaptive testing (CAT), where maintaining a consistent and meaningful scale across adaptive test administrations is crucial, could also prove fruitful.

It is worth pointing out that the bit scale does not affect model fit, and can thus be used in conjunction with most analytical methods that accompany IRT, such as differential item functioning and various item and person fit metrics (van der Linden, 2018).

In conclusion, the bit scale offers a theoretically sound and practically useful transformation for IRT scores. By grounding the latent trait metric in the information provided by the test items, it overcomes key limitations associated with the arbitrary nature of the conventional 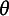 scale. Its metric properties, invariance to population assumptions, and utility in model comparison and linking suggest that bit scales can be a valuable tool for enhancing the interpretation, comparability, and evaluation of measurement outcomes in educational and psychological assessment.

## Software availability

To facilitate the application of the bit scale and encourage further research, open-source implementations have been developed for both the R and Python programming environments. For R users, the method is implemented in the package bitscale (Wallmark, 2025a). This package is designed to interface with fitted IRT models from the mirt package (Chalmers, 2012). It provides functions to compute item and test bit scores, estimate standard errors, and produce bit scale-related visualizations. The source code, installation instructions, and usage examples are available at https://github.com/joakimwallmark/bitscale.

For Python users, the bit scale is natively supported in the IRTorch package (Wallmark, 2025b). IRTorch is a standalone IRT package that leverages PyTorch for automatic differentiation and model estimation, making it suitable for modern machine learning workflows. The bit scale transformation is available as a rescaling method within the package’s API. Comprehensive documentation and tutorials can be found at https://irtorch.readthedocs.io/, and the source code is hosted at https://github.com/joakimwallmark/irtorch.
